# Glycemic control, associated factors, acute complications of Type 1 Diabetes Mellitus in children, adolescents and young adults in Tanzania

**DOI:** 10.1002/edm2.200

**Published:** 2020-11-10

**Authors:** Ronald P. McLarty, Julius P. Alloyce, Grace G. Chitema, Levina J. Msuya

**Affiliations:** ^1^ Kilimanjaro Christian Medical University College Moshi Tanzania; ^2^ Kilimanjaro Christian Medical Centre Moshi Tanzania

**Keywords:** acute complications, associated factors, glycemic control

## Abstract

**Objective:**

To determine the factors associated with poor glycemic control in children (1‐10 years), adolescents (11‐18 years) and young adults (19‐40 years) with Type 1 Diabetes Mellitus (T1DM) in Kilimanjaro Christian Medical Center (KCMC) in Moshi, Mount Meru Regional Referral Hospital (MMRRH) and Meru District Hospital (MDH) in Arusha, Tanzania.

**Methods:**

Cross sectional study of 150 participants conducted from January to June 2019, data was collected by structured questionnaire and analyzed using SPSS version 23.

**Results:**

The mean HbA1c was 12.3 ± 2.2%, 146 had poor glycemic control (HbA1c > 7.5%). BMI, insulin regime and caretaker education were associated with poor glycemic control. There were 16 participants diagnosed in DKA and the most frequently reported complications in the prior 3 months were hyperglycemia (n = 25), DKA (n = 18) and hypoglycemia (n = 4).

**Conclusions:**

Glycemic control is still very poor particularly in adolescents. Significant associations with glycemic control were higher BMI, insulin regime and guardian education. The study revealed lower prevalence of DKA at diagnosis compared to previous studies.

## Graphical abstract

A study looking at glycemic control in T1DM children, adolescents and young adults in Tanzania that reports on the proportion, riskfactors for poor glycemic control and acute complications in this same population.

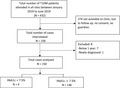

## 1 INTRODUCTION

Diabetes is a complex metabolic disorder characterized by chronic hyperglycemia resulting from defects in insulin secretion, action or both.^1^ In 2017, the International Diabetes Federation reported that 50 600 children aged 0‐19 were known to be living with type 1 diabetes mellitus (T1DM) in Africa, with about 18 300 new cases diagnosed each year.^2^ Over the past 3 decades the prevalence of diabetes has been increasing and it is growing most rapidly in low‐ and middle‐income countries (LMIC).^3^ It is estimated that there are 1.1 million children living with diabetes worldwide who are below 20 years with 132 600 being diagnosed every year corresponding to an annual increase of 3%.^4^ There are approximately 4000 children with T1DM in Tanzania who are managed in 38 clinics.

Poor glycemic control can result in microvascular complications (retinopathy, neuropathy, and nephropathy) in addition to macrovascular complications (CVA, coronary arterial disease and peripheral vascular disease). However strict glycemic control can prevent these same complications though there may be a risk of hypoglycemia.^5^

Few studies have been done looking at the cause of poor glycemic control in Sub‐Saharan African countries, hence knowing the causes of poor glycemic control would assist us to improve diabetes care by addressing and targeting these underlying causes.

## 2 METHODS

This cross sectional study conducted from January to June 2019 looked at the factors associated with poor glycemic control.

### 2.1 Subjects

T1DM study participants who totaled 432 and were registered at the following study sites: KCMC with 231, MMRRH 173 and MDH with 45.

### 2.2 Selection of participants

**FIGURE 1:**
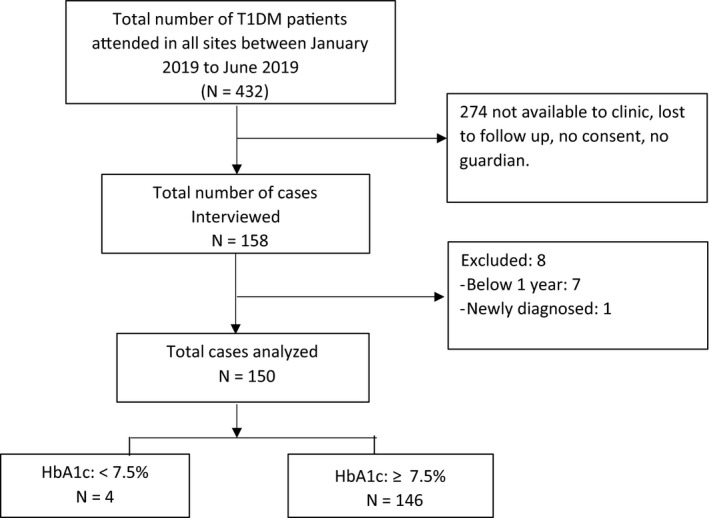
Flow chart of the participants. A total of 432 presented to study sites during the study period, a total of 274 not interviewed, most (N = 146) had poor glycemic control and only 4 presented with good glycemic control

Convenience sampling and a total of 274 participants were excluded either because they were at boarding school, lost to follow‐up or the caretaker was unavailable to give consent to study participation. Therefore a total of 158 subjects were interviewed and among these 8 did not meet the study criteria and were excluded from the analysis. The remaining 150 participants were recruited by convenience sampling and were followed up over the six month period (Figure 1).

### 2.3 Inclusion criteria

The research subjects were all attending T1DM clinics in KCMC, MMRRH and MDH.

### 2.4 Exclusion criteria

Those who did not consent to take part in the study and were aged less than 1 year.

### 2.5 Measurements

Sample size was calculated using the following formula n = (*Z* ^2^ *p* (1–*p*))/*ε* ^2^, this gives an estimated minimum sample size of 158.

The dependent variable was glycemic control and numerous independent variables were assessed including sociodemographic, clinical and diabetes related variables.

Data was collected during a short oral interview and a questionnaire consisting of 38 questions which had been translated into the Kiswahili language and were filled out by the participant and caretaker if participant was a child or adolescent or only by the participant if he or she was a young adult. 10 questions were assigned to sociodemographic details, 30 questions were clinical or diabetes related, 1 question each was assigned to diet and exercise and 3 questions concerned psychological factors in relation to T1DM. The participants were then taken to a separate room where they received a full explanation of the research and the aims. Filling of the questionnaire was assisted by the principal investigator and two research assistants with an interview duration of approximately 30 minutes. HbA1c levels were measured and recorded to assess the average glycemic control in the previous 3 months.

HbA1c levels were determined by using a DCA HbA1c analyzer and the same machine was used in MMRRH and MDH whereas a separate machine was used at KCMC (DCA 2000+). The machines were calibrated and reagents, with expiry date 2020, were type DCA 2003 Hemoglobin A1c (Siemens Healthcare Diagnostics, Benedict Avenue, New York).

### 2.6 Data analysis

Data were analyzed using SPSS version 23, numerical variables were summarized using measures of central tendency (mean + SD, median + IQR). Categorical variables were summarized using percentages and frequencies, multivariate models (logistic regression and multiple logistic regressions) were used to check for factors associated with glycemic control and a *P*‐value < .05 was considered statistically significant.

## 3 RESULTS

### 3.1 Socio‐demographic characteristics of the study participants

Table 1 shows the socio‐demographic characteristics of the study participants. Of the 150 participants enrolled in the study, the mean age was 16 years with standard deviation of 5.9 years, 74 of the participants were male and 79 had at least achieved a secondary education. A high proportion (n = 88) of the participants were from MMRRH followed by 55 from KCMC and 7 were from MDH. The average time to reach each facility was 2 hours by walking with inter‐quartile range 1 to 2 hours and the primary care giver most frequently reported was the mother that accounted for 80 of the participants, followed by fathers (n = 63) of the participants and only 7 reported having another primary caretaker. The majority of the caretakers (n = 91) had achieved a primary education and most (n = 129) were unemployed.

**TABLE 1:** Socio‐demographic characteristics of the study participants

Variables	Categories	Frequency	Percentage
Age in years	Mean, [SD]	16, [5.9]	
	<10	25	16.7
	11‐18	79	52.7
	>19	46	30.7
Sex of children	Male	74	49.3
	Female	76	50.7
Education of a child	Below primary	14	9.3
	Primary	57	38.0
	Secondary/above	79	52.7
Clinic	Mount Meru	88	58.7
	Meru	7	4.7
	KCMC	55	36.7
Time to clinic	Median, [IQR]	2, [1‐2]	
	<2 h	79	52.7
	≥2 h	71	47.3
Residence	Urban	76	50.7
	Rural	74	49.3
Primary care giver	Father	63	42.0
	Mother	80	53.3
	Others	7	4.7
Guardian education	≤Primary	91	60.7
	Secondary	40	26.7
	College/above	19	12.7
Guardian Occupation	Unemployed	129	86.0
	Employed	21	14.0

Note

Values are given as both absolute numbers and percentages, (N = 150).

Abbreviation: IQR, interquartile range.

### 3.2 Clinical and diabetes specific characteristics of the participants

Regarding clinical and diabetes related characteristics, about 25 were obese, 12 overweight, 61 had normal weight and 52 were underweight. The average duration of T1DM was 1 year and 3 months with inter‐quartile range 1 to 2 years. The majority (n = 89) of the participants received up‐to 2 insulin injections per day and most 144 used actrapid and insulatard insulin injections. 65 received insulin dose <0.8 units/kg, 59 used 0.9‐1.2 units/kg and 26 used > 1.2 units/kg. About two thirds (111) reported never missing insulin injections and history of stigmatization was less reported by the participants, as only 39 were reported feeling stigmatized (Table 2).

**TABLE 2:** Clinical and diabetic specific characteristics of the participants

Variables	Categories	Frequency	Percent
BMI	Underweight	52	34.7
	Normal weight	61	40.7
	Overweight	12	8.0
	Obese	25	16.7
Duration of diabetes (years)	Median, [IQR]	1.3, [1‐2]	
	≤5	89	59.3
	>5	61	40.7
Number of Insulin Injections	0 to 2	83	55.3
Daily	>2	67	44.7
Type of insulin	Soluble and Insulatard	144	96.0
	Others	6	4.0
Insulin dose	<0.8 units/kg	65	43.3
	0.9‐1.2 units/kg	59	39.3
	1.2 units/kg	26	17.3
Missed injections	None	111	74.0
	Once	10	6.7
	Two or more	29	19.3
Carbohydrates intake	Less	34	22.7
	Moderate	47	31.3
	Frequent	69	46.0
Exercise in the 3 mo	Stopped	124	82.7
	Few times	6	4.0
	All times	8	5.3
Stigmatized in last 3 mo	No	111	74.0
	Yes	39	26.0

Note

Values are given as absolute numbers and percentages, (N = 150).

Abbreviation: IQR, Interquartile range.

### 3.3 Proportion of children with poor glycaemic control

Most of the participants (n = 146) had poor glycaemic control (HbA1c > 7.5%) and the overall average HbA1c was 12.3% with standard deviation of 2.2% which indicates that a large group of the participants had poor glycaemic control.

### 3.4 Socio‐demographic factors associated with poor glycemic control

Children aged below 10 years had good glycemic control when compared to adolescents and young adults which was statistically significant *P* = .007 in univariate analysis. Adolescents were more likely to have poor glycemic control when compared to other groups, (HbA1c 12.8%), which was even higher than the overall mean HbA1c. The T1DM participants of caretakers who had achieved a college education and above had better HbA1c compared to the T1DM participants of less educated caretakers and this was statistically significant *P* = .028. (Table 3).

**TABLE 3:** Socio‐demographic factors associated with poor glycaemic control

Variables	Categories	N	HbA1c	Significance test	
			Mean ± SD	95% CI	*P*‐value
Overall	All	150	12.3 ± 2.2	11.9‐12.7	
Age in years	<10	25	11.6 ± 2.22	10.7‐12.5	.007
	11‐18	79	12.8 ± 2.0	12.4‐13.3	
	>19	46	11.8 ± 2.3	11.1‐12.5	
Education of a child	Below primary	14	11.6 ± 2.0	10.4‐12.7	.057
	Primary	57	12.8 ± 1.8	12.3‐13.3	
	Secondary/above	79	12.1 ± 2.4	11.6‐12.6	
Primary care giver	Father	63	12.2 ± 2.2	11.7‐12.8	
	Mother	80	12.4 ± 2.1	11.9‐12.9	.941
	Others	7	12.3 ± 2.5	10.1‐14.6	
Guardian education	≤Primary	91	12.6 ± 2.1	12.2‐13.0	.083
	Secondary	40	12.1 ± 2.3	11.4‐12.8	
	College/above	19	11.4 ± 2.2	10.4‐12.5	

Note

N = absolute number = 150, statistical significance considered at *P* < .05, hence only age was statistically significant.

Abbreviations: CI, confidence interval; SD, standard deviation.

### 3.5 Clinical and diabetes specific factors associated with poor glycemic control

Overweight participants had significantly better glycemic control when compared to the other BMI groups, and this was statistically significant *P* = .025. Insulin regime was associated with glycemic control and the result was statistically significant, those who had actrapid and insulatard had better glycemic control (HbA1c, 12.3 ± 2.2%) when compared to those who had other insulin regimens (mixed or only soluble), (HbA1c 14%); (see Table 4).

**TABLE 4:** Clinical and diabetes specific factors associated with poor glycaemic control

Variables	Categories	N	HbA1c	Significance test	
			Mean ± SD	95% CI	*P*‐value
Overall	All	150	12.3 ± 2.2	11.9‐12.7	
BMI	Underweight	52	12.7 ± 2.0	12.1‐13.3	.025
	Normal weight	61	12.5 ± 2.1	12.0‐13.0	
	Overweight	12	10.7 ± 2.7	9.0‐12.4	
	Obesity	25	12.0 ± 2.2	11.1‐12.6	
Duration of diabetes	≤5	89	12.5 ± 2.1	12.0‐13.0	.352
	>5	61	12.1 ± 2.3	11.5‐12.7	
Type of insulin	actrapid and insulatard	144	12.3 ± 2.2	11.9‐12.6	.055
	Other	6	14.0 ± 0.0	14.0‐14.0	
Insulin dose	<0.8 units/kg	65	12.3 ± 2.2	11.8‐12.8	
	0.9‐1.2 units/kg	59	12.3 ± 2.1	11.7‐12.8	.944
	>1.2 units/kg	26	12.5 ± 2.5	11.5‐13.5	

Note

N = absolute number = 150, *P* value < .05 considered statistically significant.

Abbreviations: CI, confidence interval; SD, standard deviation.

In the final model, socio‐demographic, clinical and diabetes mellitus related factors with p value <0.1 in the analysis of variance were run using multivariate regression analysis to control for possible confounders and modifiable effects and to study their significance risk on glycemic control. The result indicated that higher BMI, type of insulin (actrapid and insulatard) and guardian education were significantly associated with better glycemic control, *P* < .05. (See Table 5).

**TABLE 5:** Multivariate regression analysis for the risk factors associated with poor glycaemic control

Variables	Unstandardized Coefficients		Standardized Coefficients		95% CI	*P*‐value
	Beta	SE	Beta	*t*		
Age	0.31	0.35	0.10	0.9	−0.37, 1.00	.367
BMI	−0.41	0.19	−0.20	−2.12	−0.80, −0.03	.035
Participant Education	−0.11	0.32	−0.03	−0.32	−0.75, 0.54	.745
Guardian education	−0.57	0.25	−0.19	−2.22	−1.06, −0.06	.028
Type of insulin	2.15	0.90	0.19	2.38	0.37, 3.93	.018

Note

Multivariate regression analysis for the risk factors associated with poor glycemic control, (N = 150). *P* value < .05 considered statistically significant.

Abbreviations: CI, confidence interval; SE, standard error.

### 3.6 Acute complications among T1DM children, adolescents and young adults

**FIGURE 2:**
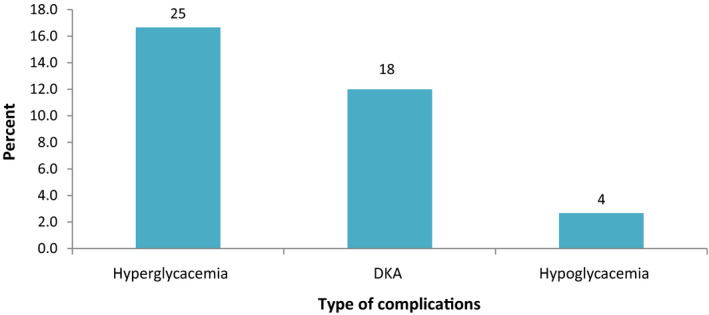
The acute complications in T1DM among participants. (N = 150), acute complications were as reported in the last 3 months. Only absolute numbers are reported

**FIGURE 3:**
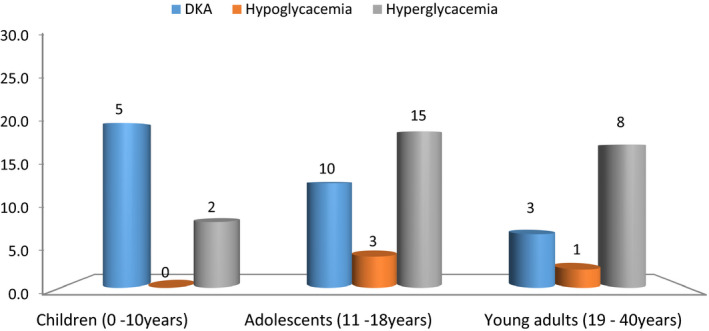
Acute complications related to T1DM by age group of the study participant. (N = 150), Complications were as reported in the last 3 months, only absolute numbers are reported

There were 16 participants who presented with DKA at diagnosis of T1DM, 47 T1DM participants reported to have acute complications related to T1DM in the last 3 months, the most frequently reported complications in the preceding three months were hyperglycemia (n = 25) followed by DKA (n = 18) and hypoglycemia (n = 4), (Figure 2). Hyperglycemia and hypoglycemia were more prevalent in adolescents and young adults, whilst DKA was more commonly reported in children (Figure 3).

## 4 DISCUSSION

The key finding from this study was that 146 study participants had poor glycemic control (HbA1c > 7.5%) with a mean HbA1c was 12.3 ± 2.2%. Factors associated with glycemic control were BMI, insulin type and caretaker educational achievement and it was found that the incidence of DKA was only 10.7% at diagnosis of T1DM.

The finding that the mean HbA1c is 12.3 ± 2.2% is similar to a Kenyan study by Ngwiri et al^5^ where the median HbA1c was 12.3% and also a Tanzanian study by Mukama et al^6^ where the mean HbA1c was >12.3%. This finding is in contrast to the tightly controlled studies in the developed world where there are prosperous economies and intensive approach to diabetes management for example DCCT, 1982‐93^4^ where HbA1c was <7% was achieved. In this study the lowest HbA1c was found in the overweight BMI group (n = 12), Hba1c 10.7 ± 2.7% though the majority (n = 61) of the participants were normal weight and this was statistically significant. This was similar to the SEARCH for diabetes in youth study which revealed worse glycemic control in T1DM youths who were underweight (<85th Percentile) or normal weight compared to obese subjects^7^). The findings are not dissimilar to a Romanian study of 662 T1DM patients^8^ which revealed that a BMI above the target (>25 kg/m^2^) was not associated with greater odds of having an HbA1c higher than the standard (>7%) compared to individuals with normal BMI. The reason for this may be that those with a higher BMI have less severe metabolic decompensation and hence easier and less difficult to control diabetes unlike those who are underweight and have absolute loss of insulin which results in catabolic state and significant weight loss. A possible implication of this finding could be a lower insulin requirement in overweight patients and this would benefit Tanzanian T1DM patients who have been reported to “reduce the prescribed insulin dose in order to guarantee a longer period of treatment.”^9^ Insulin regime used was significantly associated with HbA1c since those participants who were using actrapid and insulatard in separate injections (n = 144) had an HbA1c of 12.3 ± 2.2% whilst those using other insulin combinations (n = 6) such as mixtard or only soluble had HbA1c 14%. These findings were identical to the findings in a study by Mukama et al^6^ though in his study the results were not statistically significant (.455). An implication of this finding is that separate insulin allows for more mealtime flexibility over mixed insulin and hence may be suitable for Type 1 diabetes mellitus patients in Tanzania who have been shown to have problems with regular meals.

Caretaker education is significantly associated with HbA1c and the results showed that most (n = 91) of the caretakers had only achieved a primary level education and this was associated with the highest HbA1c (12.6 ± 2.1%). The level of HbA1c declines with increasing level of educational attainment, (12.1 ± 2.3%) for secondary school level and (11.4 ± 2.4%) for college level education and beyond. Literature has shown that parent's education level plays a major role in the glycemic control of a child with T1DM, for instance a study conducted in Saudi Arabia by A AlAgha et al^10^ revealed that the children of more educated fathers had better glycemic control (HbA1c < 7%) whilst poor glycemic control was found in children of low educated fathers but there was no significant differences between HbA1c and mothers education level. The implication of this finding is that there is a need for continuing caretaker education about T1DM regardless of educational achievement but particularly in those with low educational achievement. Those with lower educational achievement may be less likely to understand aspects of diabetes management including but not limited to education about insulin therapy, diet, blood glucose monitoring and physical activity in T1DM. Hence education materials should also be adapted to populations with poor reading skills.

There were 16 participants who presented with DKA at diagnosis of T1DM (10.7% of study participants), and this was in contrast to previous studies by Majaliwa et al and Mukama et al^6^, ^9^ who found that 89.9% and 86% of participants respectively presented with DKA. The finding in this study might be a reflection of the non‐biochemical definition of DKA which was used in this study based on self‐reporting of symptoms rather than a strictly biochemical definition. It may also be a reflection of the improved ability of the patients and caretakers to not only manage sick days but also detect the symptoms of DKA hence reducing the need for hospital admission due to DKA. Another reason for the low incidence may be as a result of good teaching or diabetes mellitus specific education dispensed at clinic level over the years.

After completion of this research project the question might be posed; what can be done to address the barriers to glycemic control? The answer to the problem of poor glycemic control is likely found in structured diabetes education but also addressing the barriers to achieving glycemic targets as outlined by Steyn et al^11^ which might include, regarding funding for diabetes: governments putting more emphasis on communicable disease than non‐communicable disease. Regarding research: research gap due to financial constraints, lack of aggressiveness of pediatric diabetes researchers to advocate for diabetes research and shortcomings in diabetes research dissemination. Regarding diagnosis: lack of awareness about the disease, majority undiagnosed, poor screening and surveillance, lack of infrastructure. Regarding treatments: few T1DM specialists, lack of access, availability and affordability of insulin, lack of education directed at children, families and communities about T1DM and lack of treatment referrals. Improved methods to more effectively monitor blood glucose include possibility of using continuous glucose monitoring devices as has been trialed by Yauch et al^12^ in Uganda and Kenya, March 2020. The study revealed that CGM is feasible and well accepted in patients in resource poor settings. Another target area for improving glycemic control is insulin, not only access but also storage. East African Diabetes Study Group Guidelines recommend insulin storage in a clean container in a cupboard at room temperature. Finally, in East Africa children have little access to snacks between meals and they have high activity levels and limited access to between meal carbohydrates puts these children at risk of hypo‐glycaemia when the morning (NPH insulin peaks).

## 5 STRENGTHS AND LIMITATIONS

There are limitations in the use of HbA1c as an assessment tool for assessing glycemic control as it is affected by haemoglobinopathies, certain forms of anemia or other conditions affecting turnover of red blood cells (hemolysis and iron deficiency) which were not screened for in our study.

This study is one of the few studies assessing glycemic control in East Africa in children, adolescents but also inclusive of young adults. Most prior studies only include children and adolescents which is not representative of the type 1 diabetes mellitus population served by the clinics.

This study included a sample size of 150 which differs from other previous studies performed in the region in which the sample size ranges from 41 to 104 participants, this increases the power and statistical validity of the study.

## 6 CONCLUSIONS AND RECOMMENDATION

Glycemic control in children, adolescents and young adults with T1DM attending these clinics is still very poor and the factors associated with glycemic control from this study were BMI, insulin type and guardian education achievement. There is a lower incidence of DKA at diagnosis compared to previous studies and this may reflective of the good quality T1DM related education provided over the years. Further recommendations are the introduction of participant led peer to peer counselling in collaboration with the medical professionals which has recently been established at one clinic in Dar es Salaam, Tanzania. An additional recommendation would be to set up separate male and female adolescent discussion groups.

Specific actions could be taken to improve diabetes education might include improving overall literacy levels, regular diabetes camps where there can be support and educational activities, education that focusses on prevention as well as treatment, with materials that are culturally appropriate and adapted to populations with poor reading skills.

Finally the national policy framework for non‐communicable diseases and diabetes should incorporate prevention, organization of care, education, disease monitoring, and allocation of appropriate resources. Strategies to improve overall management of T1DM in African children, as outlined by Steyn et al^11^ would include the following: ensuring a minimum standard of care for T1DM, ensuring insulin is available always and in sufficient quantities to meet the needs of those with T1DM. Children and their families should have access to comprehensive diabetes care which includes treatment, monitoring psychosocial support. There should be a sufficient number of healthcare staff having a specialization in diabetes, should be trained in order to meet diabetic care needs in both urban and rural health facilities. Ongoing education is essential at multiple levels is required to educate children, their families and the broader public. Additionally the following could be considered,ensuring that all T1DM patients are covered by the national health insurance scheme, multidisciplinary approach to type 1 diabetes mellitus management, improving infrastructure and supply of medical and laboratory equipment and increasing the number of African pediatricians trained in pediatric endocrinology.

## CONFLICT OF INTEREST

The author declares no financial relationships or conflicts of interest in relation to this article.

## AUTHOR CONTRIBUTIONS

RPM designed the study, performed data entry, contributed to discussion, wrote the manuscript and reviewed and edited the manuscript. JAA reviewed the data, assisted with data cleaning and analysis, reviewed the manuscript. GGC assisted with data collection, assisted in calling participants to remedy missing data, assisted with measuring participant HbA1c levels. Professor LJM, D.M and Professor P.C reviewed and edited the manuscript.

## ETHICAL APPROVAL

Ethical approval and consent to participate: consent for the study was obtained from the Kilimanjaro Christian Medical University College ethics committee with certificate number 2100. Consent for study participation was also obtained from the participants and caretakers.

## Data Availability

The datasets used and/or analyzed during the current study are available from the corresponding author on reasonable request.
